# Circular RNA Circ_0005564 promotes osteogenic differentiation of bone marrow mesenchymal cells in osteoporosis

**DOI:** 10.1080/21655979.2021.1959865

**Published:** 2021-08-10

**Authors:** Zitao Liu, Qiyu Liu, Shanchuang Chen, Haitao Su, Tao Jiang

**Affiliations:** Department of Orthopaedics, The Second Affiliated Hospital of Guangzhou University of Chinese Medicine, Guangzhou, Guangdong, China

**Keywords:** Circ_0005564, osteogenic differentiation, BMSCs, high-throughput sequencing

## Abstract

Circular RNA **(C**ircRNA) plays a potential role in bone formation. We aimed to study the circRNAs expression profiles and their functions in osteogenic differentiation of human bone marrow stromal cells (BMSCs). Firstly, we established osteogenic differentiation of BMSCs displaying increased mRNA expression of osteogenic differentiation marker (RUNX2, OPN, and OCN), increased ALP activity and protein expression, and increased mineralized nodules formation, as well as morphological alteration. Then, we employed high-throughput sequencing to analyze circRNA expression and found that 3440 and 3893 circRNAs in non-induced and induced groups, respectively. We further validated the 10 differentially expressed circRNAs with the most significant difference between induced and non-induced groups. Among these ten circRNAs, five of them with more than one miRNA binding site were used to construct a ceRNA network exhibiting 81 miRNAs and 182 target mRNAs. Furthermore, among these five circRNAs, we found only circ_0005564 significantly reduced the mRNA expression of RUNX2, OPN, and OCN. The circularity of circ_0005564 was verified. Our results showed that knockdown of circ_0005564 inhibited osteoblast differentiation in BMSCs. Taken together, our study demonstrates that circ_0005564 is a potential positive regulator of osteogenic differentiation of BMSCs.

## 1. Introduction

Osteoporosis (OP) is a disease characterized by impaired bone strength. OP patients have a higher risk of fractures in the hip, spine, and other body parts. Among the elderly, especially menopausal women, OP is particularly prevalent [1]. OP results in heavy economic burdens; the cost in the United States and the United Kingdom is about 17.9 US dollars/year and 4 billion pounds/year, respectively [2]. Although current osteoporosis drugs can significantly reduce fractures, the prevention of OS and investigation of new therapeutic targets remain essential.

Normal bone turnover depends on the balance between bone resorption and bone formation. In high-risk patients with OP, such as menopausal women, this balance is usually broken; that is, bone formation cannot supplement bone resorption bone mass, thus showing the characteristics of progressive bone loss [3]. Therefore, promoting bone formation may be the key to improving OP’s bone loss, and it is of great significance to study the key factors regulating bone formation.

Circular RNAs (circRNAs) are special non-coding RNAs that widely exist in various organisms [4,5]. Distinct from linear RNA, the shape of circRNAs is head-to-tail, regulating gene expression by sponging miRNAs to compete with mRNA for miRNAs binding [6]. CircRNAs regulates a series of biological processes, such as tumorigenesis, diabetes, and cartilage degradation process [6–8]. Recent studies have reported that several circRNAs were differentially expressed in menopausal women, suggesting that circRNAs are involved in regulating menopausal-related pathogenesis [9]. Besides, circRNAs are involved in bone formation [10]. Yin et al. found that CircRUNX2 served as a hsa-miR-203 sponge to enhance runt-related transcription factor 2 (RUNX2) expression and promoted bone formation [11]. Osteocalcin (OCN) and osteopontin (OPN) are the well-known markers for the late-stage osteogenic differentiation [12,13]. RUNX2 is considered a critical target for osteogenic differentiation and regulates its downstream molecules, including OCN and OPN [14]. Decreased expression of Circ-VANGL1 promoted the miRNA-217 expression in OP patients and thereby negatively regulated RUNX2 expression as well as the expression of bone sialoprotein (BSP), osteocalcin (OCN), and osteopontin (OPN) to inhibit the differentiation of BMSC [15]. However, it remains largely unknown about the role of circRNAs playing in osteogenic differentiation.

We hypothesized that circRNAs contribute to the osteogenic differentiation of BMSCs. This study aimed to investigate the differentially expressed circRNA profiles and their functions in osteogenic differentiation of BMSCs. In this study, we employed high-throughput sequencing to analyze the expression profiles of circRNAs during the osteogenic differentiation of BMSCs and analyze the function of differentially expressed circRNAs. The goals of this study are to provide insights into the disease mechanism of OP and potential therapeutic targets for the treatment of OP.

## 2. Methods

### 2.1. BMSCs osteogenic differentiation induction

BMSCs were purchased from Guangzhou Saliai Stem Cell Science and Technology (Guangzhou, China) and cultured in a-MEM medium with 10% fetal bovine serum, 1% glutamine, and 1% penicillin-streptomycin. BMSCs seeded into 6-well plates and were divided into osteogenic differentiation induced group (Ind group) and non-induced group (Non-ind). The Ind group was treated with OriCell human mesenchymal stem cell osteogenic differentiation medium kit (Cyagen, China, catalog number: HUXMA-90,021) for 14 days according to the manufacturer’s instructions [16]. Cells were collected at the 3^rd^ day, 7^th^ day, and 14^th^ days after induction for subsequent indicated experiments, respectively.

### 2.2. Alizarin red staining

Alizarin Red staining was carried out as previously described [17]. After inducing osteogenesis differentiation for 14 days, the differentiation medium was discarded, and BMSCs were washed with PBS twice. BMSCs were then fixed with 2 ml 4% formaldehyde in each well for 30 min. Then, cells were washed with PBS twice and added 1 ml 0.2% alizarin red solution (Solarbio, China) in each well and stained for 5 min. Osteogenic staining images were captured under a microscope after washing with PBS.

### 2.3. ALP staining and ALP activity measurement

ALP staining solution including Naphthol AS-MX Phosphate (Sigma N-4875), N, N-Dimethylformamide (DMF), N, N-Dimethylformamide (DMF), 0.2 M Tris-HCl Buffer (pH = 8.5), and distilled water was prepared immediately before use. We added ALP staining solution and incubated it in the dark for 1 h on a shaker. ALP staining images were taken under a microscope.

ALP activity was determined using an alkaline phosphatase assay kit (Nanjing Jiancheng Bioengineering Institute, China) following manufacturer’s instructions. The absorbance was read on a multiscan GO system (ThermoFisher, USA).

### 2.4. RNA sequencing

Total RNAs were isolated from both groups after inducing for 14 days using TRizol reagent (Thermo Scientific, MA, USA). Ribosomal RNAs (rRNAs) and linear RNAs were removed from total RNA by magnetic bead selection and digesting with RNase R (Epicenter, Paris, France). Digested RNAs were broken into short RNA fragments and used for first-strand and second strand cDNA synthesis. The purified double stranded cDNAs were purified by AgencourtAMPure XP beads (Beckman coulter life sciences, Indiana, the USA). We added Poly-A tail after repairing the end of double stranded cDNA, and the cDNAs were then ligating to ‘U-adaptor’. PCR amplification was used to purify the beads and then AgencourtAMPure XP beads (Beckman coulter life sciences, Indiana, the USA) were used for fragment size selection. After that, the first strand of cDNA containing U was degraded by USER enzyme (New England Biolabs, MA, USA), and the strand-specific cDNA library was enriched by PCR. cDNA library was determined by Agilent 2100 and quantified by qPCR, following by sequencing using The Illumina HiSeq platform 2500 (San Diego, CA, USA) with paired-end 150 bp (PE150) reads and a sequencing depth of 15 GB per sample.

### 2.5. Bioinformatic analysis

Raw reads obtained by the sequencing platform are subjected to data quality control (QC). High-quality clean tags were obtained by removing microRNA (miRNA), ribosomal RNA (rRNA), transfer RNA (Trna), small nuclear RNA (), Small nucleolar RNA (snoRNA), exon, intron, etc. CircRNA with a p < 0.01 and a fold change >2 was defined as differentially expressed circRNA. Target miRNAs and RNA binding proteins (RBPs) of circRNAs were predicted on the website of miRbase (http://www.mirbase.org/) [18–23] and CircInteractome (https://circinteractome.irp.nia.nih.gov/) [24]. Gene Ontology (GO) and Kyoto Encyclopedia of Genes Genomes (KEGG) pathways enrichment analyses of differential expressed circRNAs were performed using R package v3.4.2. circRNA-miRNA-mRNA network was drawn by Cytoscape v3.7.2. Gene set enrichment analysis (GSEA) was performed in GSEA software (v4.1.0) to analyze parent genes of differentially expressed circRNAs in osteogenic differentiation-related pathways [25,26]. Nominal p-value <0.05 was considered significantly enriched.

### 2.6. Quantitative reverse transcription-polymerase chain reaction (qRT-PCR)

BMSCs were collected after treatment. Total RNAs were isolated using TRIzol (Invitrogen, USA) and used for synthesizing cDNAs using a cDNA Reverse Transcription Kit (Takara, Tokyo, Japan). The qRT-PCR analysis was performed using the LightCycler 480 SYBR Green I Master kit on a LightCycler 480 II (Roche) under the following conditions: initial denaturation at 95°C for a 5 min, followed by 45 cycles at 94°C for 10 s and 60°C for 30 s, a final extension at 72°C for 30 s. Convergent primers were designed to detect RUNX2, OPN, OCN, and FGFR1. Divergent primers were designed for identify the expression of circRNA (circ_0109177, circ_0001085, circ_0005078, circ_0002075, circ_0002346, circ_0006083, circ_0006482, circ_0000707, circ_0005564 and circ_0005255). Convergent primers and divergent primers for PCR reaction of circ_0005564 and GAPDH were used to verify the circular form of circRNAs via agarose gel electrophoresis, followed by sanger sequencing of products from circ_0005564 PCR using divergent primers. The relative expression of genes was evaluated by the comparative threshold cycle method. GAPDH was used as an internal control to normalize the level of osteogenesis differentiation markers and circRNAs. Primers’ sequences are shown in Table 1. The experiment was performed independently three times.

**Table 1.** Primers for osteogenesis differentiation markers and circRNAs

circRNAs	Forward (5ʹ-3ʹ)	Reverse(3ʹ-5ʹ)
RUNX2	TGGTTACTGTCATGGCGGGTA	TCTCAGATCGTTGAACCTTGCTA
OPN	AGACCCTTCCAAGTAAGTCC	TCATCTACATCATCAGAGTCGT
OCN	CGCTACCTGTATCAATGGCTGG	ATGTGGTCAGCCAACTCGTCA
GAPDH-c	GAGTCAACGGATTTGGTCGT	GAGTCAACGGATTTGGTCGT
GAPDH-d	TCCTCACAGTTGCCATGTAGACCC	TGCGGGCTCAATTTATAGAAACCGGG
FGFR1	CCCGTAGCTCCATATTGGACA	TTTGCCATTTTTCAACCAGCG
circ_0109177-d	TGGATGGACACAGAGTGCC	TGACAACGCTCCCAGGTAG
circ_0001085-d	GAAAGAGAAAGTGGAGATCGAA	TCATCAATGTGTGAGGTAAAAGAC
circ_0005078-d	AGCCCAGAAGAGCACAGAG	TATGATGGATAGAGTCTTCAATGTA
circ_0002075-d	CACCGTGTCCAAAAAGGGCT	GGGTACTCCTTGGGCCAGC
circ_0002346-d	CCTCCTGTTCAGCAGACCG	TACATTCACATTTCCCATTGATTAT
circ_0006083-d	AAGGGACAGTCAAGGTAAAGCTCC	TAAGGTGCTGGGGACAGGAGA
circ_0006482-d	TGAAGAGGCTCGGAGAAGGAC	AAACTGGAGAGACAGATTGGTTCC
circ_0000707-d	TTGAAGAGGCTCGGAGAAGGAC	GTGTTTGTCGCTGTTCTCCCTG
circ_0005564-d	CCAGTGGCTAAAGCACATCG	CAGAGGGCACCACAGAGTCC
circ_0005564-c	AAACAGTGGCCCTGGGTA	TGGACATAAGGCAGGTTGT
circ_0005255-d	ACTCCTAGCCAATGGTGGTCAT	AGTCCCGCTTTATCTCTCCCTC

### 2.7. SiRNA transfection

BMSC cells were seeded in 6-well plates for transfection. Silencing of circRNAs was performed using custom-designed siRNAs, and negative control siRNA (siNC) was set as control (sequences information referred to Table 2). 30 pmol of siRNAs and siNC were transfected in each well using Lipofectamine RNAi max (Invitrogen MA USA) according to manufacturer’s instructions.

**Table 2.** siRNAs’ sequences

Name	sense (5ʹ-3ʹ)	antisense (5ʹ-3ʹ)
circ_0006083	UACCGGGAGGCAGGACACAAU	AUUGUGUCCUGCCUCCCGGUA
circ_0006482	GGAGGAAAUGGAGGCUUUUGU	ACAAAAGCCUCCAUUUCCUCC
circ_0000707	UGCUGGCAGUAACUGGCUUUU	AAAAGCCAGUUACUGCCAGCA
circ_0005564	UCCAGAUCUUGAAGGUCCGUU	AACGGACCUUCAAGAUCUGGA
circ_0005255	GCGAUACUACAGGCAGUUGCA	TGCAACTGCCTGTAGTATCGC
siNC	UUCUCCGAACGUGUCACGUTT	ACGUGACACGUUCGGAGAATT

### 2.8. Western blot assay

BMSCs were collected and lysed using lysis buffer on ice for 10 min. The lysates were centrifuged at 12,000 g under 4°C for 10 min. Then, protein concentration in samples was measured by Bradford. Protein samples were separated by SDS-PAGE electrophoresis, followed by transferring to a nitrocellulose membrane (NCM). The NCM was then blocked in TBST containing 5% nonfat milk powder for 60 min, incubated with primary antibodies (RUNX2: CST, No. 8486; OPN: Boster, No. PB0589; OCN: Abcam, No. ab93876; and GAPDH: Santa, No.25778) overnight at 4°C, subsequently incubated with HRP labeled second antibody at room temperature for 1 h. The blotting bands were detected by ECL chemiluminescence (Santa Cruz, California, USA).

### 2.9. Statistical analysis

Data were expressed as mean ± standard deviation. Comparisons between non-ind group and Ind group were performed using unpaired Student’s t-test. Comparisons of differences among three and above groups were evaluated by One-way ANOVA analysis followed by Fisher LSD method. Statistical analyses were performed using SPSS software Version 22.0 (SPSS Inc., Chicago, IL, USA), and p < 0.05 was considered statistically significant.

## 3. Results

Osteoporosis is associated with the imbalance of bone resorption and bone formation. We hypothesized that circRNAs participate in osteogenic differentiation of BMSCs being involved in the pathogenesis of osteoporosis. We induced osteogenic differentiation in BMSCs and determined the differentially expressed circRNAs between the induced and control group through RNA sequencing. Bioinformatics analysis was performed to investigate the potential biological functions of differentially expressed circRNAs. We identified circ_0005564, an upregulated differentially expressed circRNA in BMSCs after inducing osteogenic differentiation, was a critical regulator of osteogenic differentiation of BMSCs through siRNA-mediated knockdown of circ_0005564.

### 3.1. Induction of osteogenic differentiation of BMSCs

**Figure 1.**
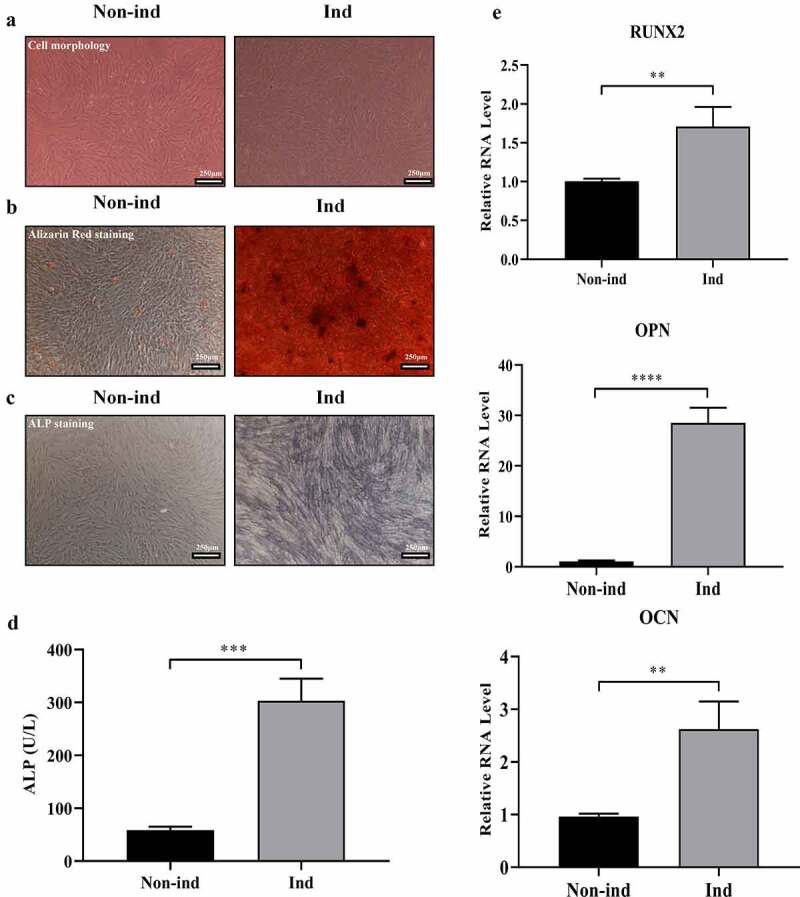
Induction of BMSCs osteogenic differentiation. (a) BMSCs’ cell morphology switched from elongated fusiform to polygonal (Magnification: 200×, scale bar: 250 μm). (b) Alizarin Red staining showed increased mineralized nodules formation (Magnification: 200×, scale bar: 250 μm). (c) ALP staining showed increased ALP protein expression (Magnification: 200×, scale bar: 250 μm). (d) ALP activity tests showed increased ALP activity (***p < 0.001). (e) qRT-PCR showed increased the mRNA expression of RUNX2, OPN, and OCN (**p < 0.01, ****p < 0.0001). Non-ind: Non-induced group; Ind: Induced group

We observed that cell morphology switched from elongated fusiform to polygonal (Figure 1(a)) and mineralized nodules increased, indicated by Alizarin Red staining (Figure 1(b)) at 14 days of osteogenic differentiation in BMSCs. Parallelly, ALP staining and ALP activity assays showed that ALP protein and its activity were significantly increased in Ind group when compared with non-ind group (Figure 1(c) & (d)). Meanwhile, mRNA expression of osteogenic differentiation markers, including RUNX2, OPN, and OCN, were significantly up-regulated in Ind group when compared with Non-ind group (Figure 1(e)). The above results demonstrate that BMSC osteogenic differentiation is successfully induced under indicated culture medium in the method section after 14 days of induction.

### 3.2. circRNAs expression profiles and function analysis in BMSCs of osteogenic differentiation

**Figure 2.**
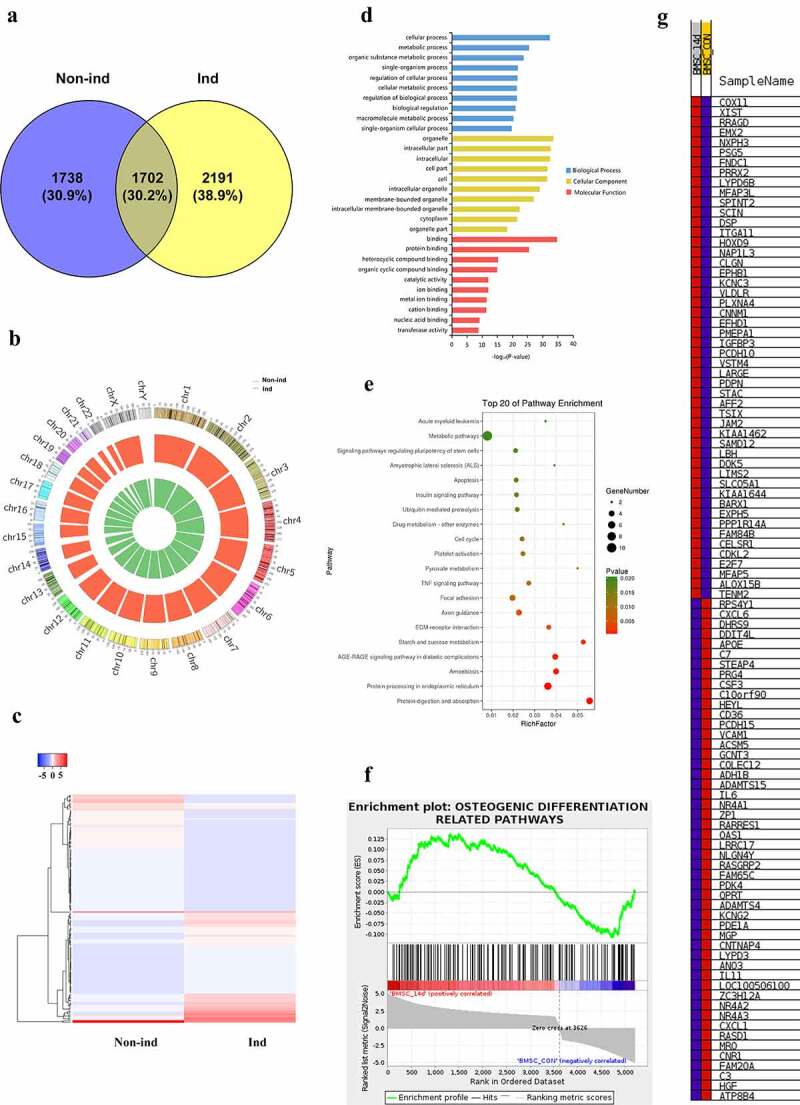
**(a)** Differentially expressed cirRNAs profile and function analysis. Venn diagram showed the number of circRNAs detected in Non-ind group and Ind group. (b) Chromosomes distribution of circRNAs. Non-ind group: inner green circle; Ind group: outer red circle. (c) Heat map of differential circRNAs in Non-ind group and Ind group. (d) GO analysis result of the parent gene of differentially expressed circRNAs in biological process, cellular component, molecular function. (e) KEGG analysis result of the parent gene of differentially expressed circRNAs. (f) GSEA analysis result of the parent genes of differentially expressed circRNAs being involved in osteogenic differentiation-related pathways. (g) Heat map of the parent genes of differentially expressed circRNAs in GSEA analysis

Next, we determined circRNAs’ expression through RNA sequencing. We identified 3440 circRNAs in non-ind group, and 3893 circRNAs in Ind group, respectively (Figure 2(a)). 1702 circRNAs overlapped in both groups (Figure 2(a)). These circRNAs were broadly distributed across the 24 pairs of human chromosomes (Figure 2(b)). The differentially expressed circRNAs were shown in a heatmap (Figure 2(c)). Further, GO analysis showed that the parent genes of differentially expressed circRNAs were involved in cellular process, metabolic process, organelle, intracellular part, and binding (Figure 2(d)). KEGG pathway analysis showed that the parent of differentially expressed circRNAs potentially participated in 20 pathways, while metabolic pathways, protein processing in the endoplasmic reticulum, and protein digestion and absorption were the most closely related signaling pathways (Figure 2(e)). Then we further performed GSEA analysis to explore the statistically significant difference of the parent of differentially expressed circRNAs involved in osteogenic differentiation-related pathways between two groups. These pathways include bone morphogenetic protein (BMP)/Smad, WNT/β-catenin, Notch, sonic hedgehog (Shh), mitogen-activated protein kinase (MAPK), and Fibroblast growth factor (FGF)/fibroblast growth factor receptor (FGFR) signaling pathways. The GSEA analysis results showed these parent genes enriched in the Ind-14d group (figure 2(f) and Figure 2(g)), indicating that osteogenic differentiation was effectively established at 14 days of induction. The parent genes being involved in osteogenic differentiation-related pathways depicted in figure 2(f) were shown in the supplementary Table S1.

### 3.3. Validation of differentially expressed circRNAs and construction of competing endogenous RNA (ceRNA) network

**Figure 3.**
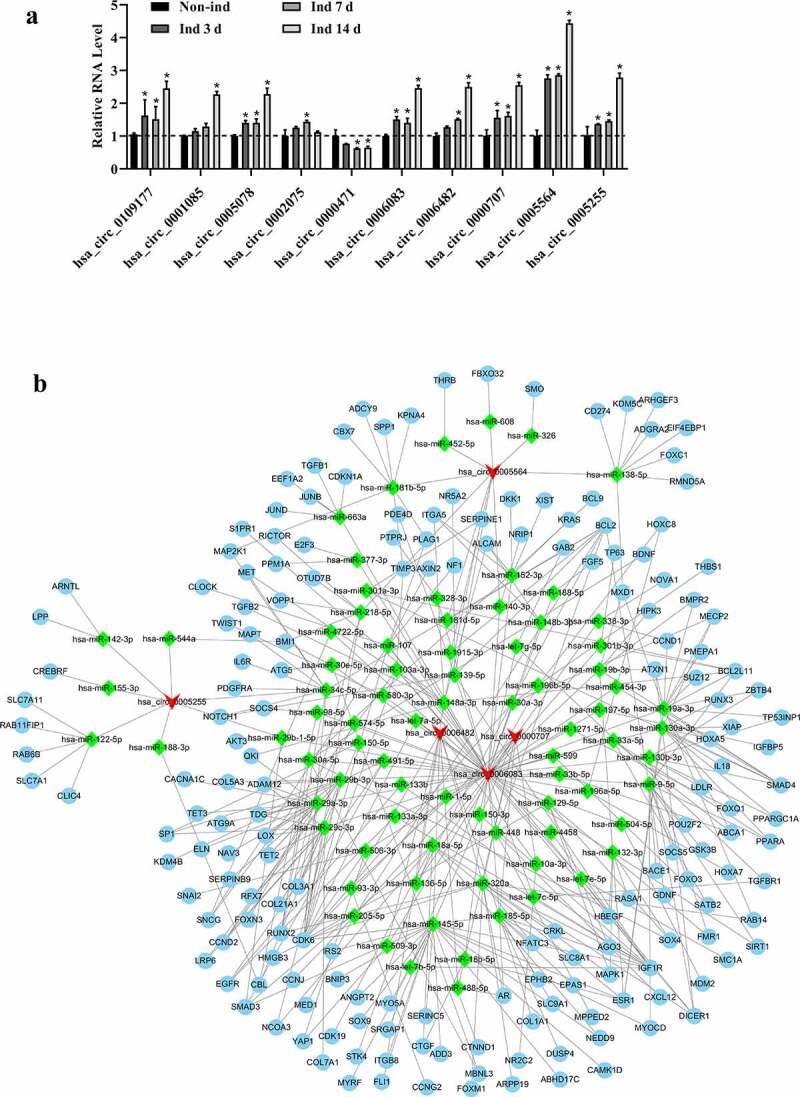
The validation of most differentially expressed circRNAs and construction of ceRNA networks (a). Circ_0109177, circ_0001085, circ_0005078, circ_0002075, circ_0000471, circ_0006083, circ_0006482, circ_0000707, circ_0005564, and circ_0005255 were determined by qRT-PCR. All experiments were performed in triplicate (*p < 0.05). (b) The ceRNA networks of five differentially expressed circRNAs with more than one binding miRNA site

Among differentially expressed cicRNAs, 10 most significant changes of differentially expressed circRNAs (Supplemental Table S4) whose parent genes are involved in regulating osteogenic differentiation were selected to verify their expression using qRT-PCR. The qRT-PCR results showed that circ_0109177, circ_0001085, circ_0005078, circ_0006083, circ_0006482, circ_0000707, circ_0005564 and circ_0005255 were up-regulated in Ind group when compared to Non-ind group. In contrast, circ_0000471 was down-regulated after inducing at the 7^th^ and 14^th^ day, respectively. However, circ_0002075 was upregulated after 7 days without a significant difference on the 14^th^ day of induction (Figure 3(a)). Based on miRbase and CircInteractome web tool searching results, five circRNAs with more than one miRNA binding site were chosen to construct a ceRNA network: 81 miRNAs and 182 target mRNAs in this network (Figure 3(b)).

### 3.4. Circ_0005564 regulated osteogenic markers expression

**Figure 4.**
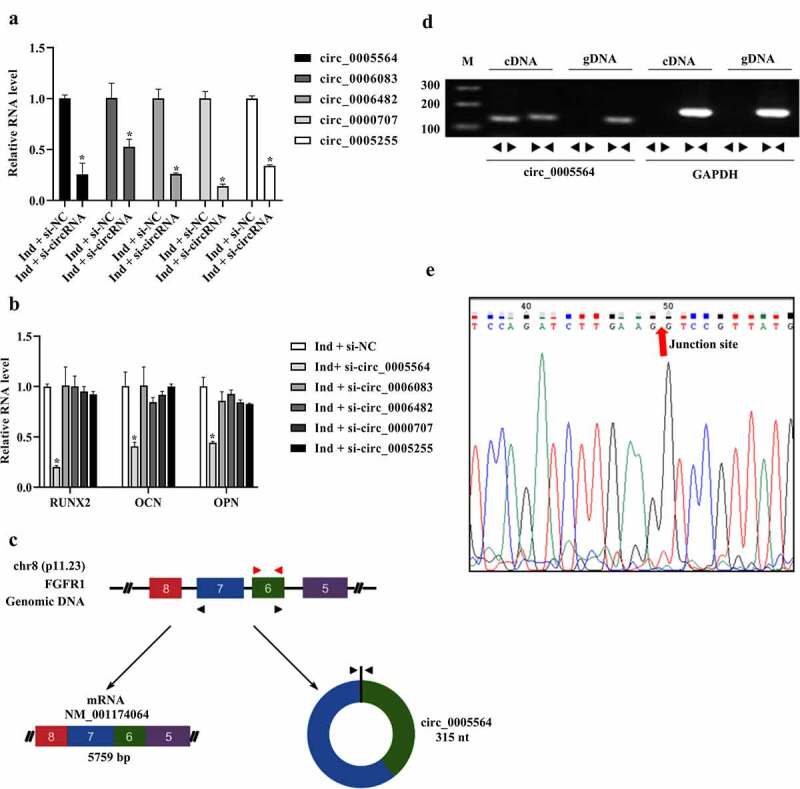
Effects of circ_0005564 on the expression of osteogenic markers. (a) siRNAs was transfected into BMSCs, and circRNAs expression was tested by qRT-PCR. *p < 0.05. (b) RUNX2, OPN, and OCN were detected by qRT-PCR after circ_0005564 silencing. *p < 0.05. (c) The formation pattern of circ_0005564. (d) Divergent and convergent primers were designed to determine the circular form of circ_0005564. PCR products were observed through agarose gel electrophoresis. (e) Sanger sequencing of products from divergent primers of circ_0005564

Next, we synthesized siRNAs of the above 5 selected circRNAs to determine their effects on BMSCs osteogenic differentiation. Our results indicated that the respective siRNAs of the selected circRNAs (circ_0005564, circ_0006083, circ_0006482, circ_0000707, and circ_0005255) effectively silenced the expression of corresponding circRNAs (Figure 4(a)). However, the expression of osteogenic markers (RUNX2, OPN, and OCN) was only significantly reduced after circ_0005564 knockdown. Thus, we focused on circ_0005564 in the subsequent experiments (Figure 4(b)). As shown in Figure 4(c), circ_0005564 generates from chromosome 8, the exon 6 and 7. The circularity of circ_0005564 was confirmed by determining the PCR production of complementary DNA (cDNA) and genomic DNA (gDNA). Our result showed the circ_0005564 was amplified with divergent primers in cDNA but not gDNA, circ_0005564 parent gene was amplified with convergent primers in cDNA and gDNA (Figure 4(d)). Further sanger sequencing of products from divergent primers of circ_0005564 showed the junction site of circ_0005564 (Figure 4(e)), indicating that the circ_0005564 is circular.

### 3.5. Circ_0005564 silencing inhibited BMSCs osteoblast differentiation

**Figure 5.**
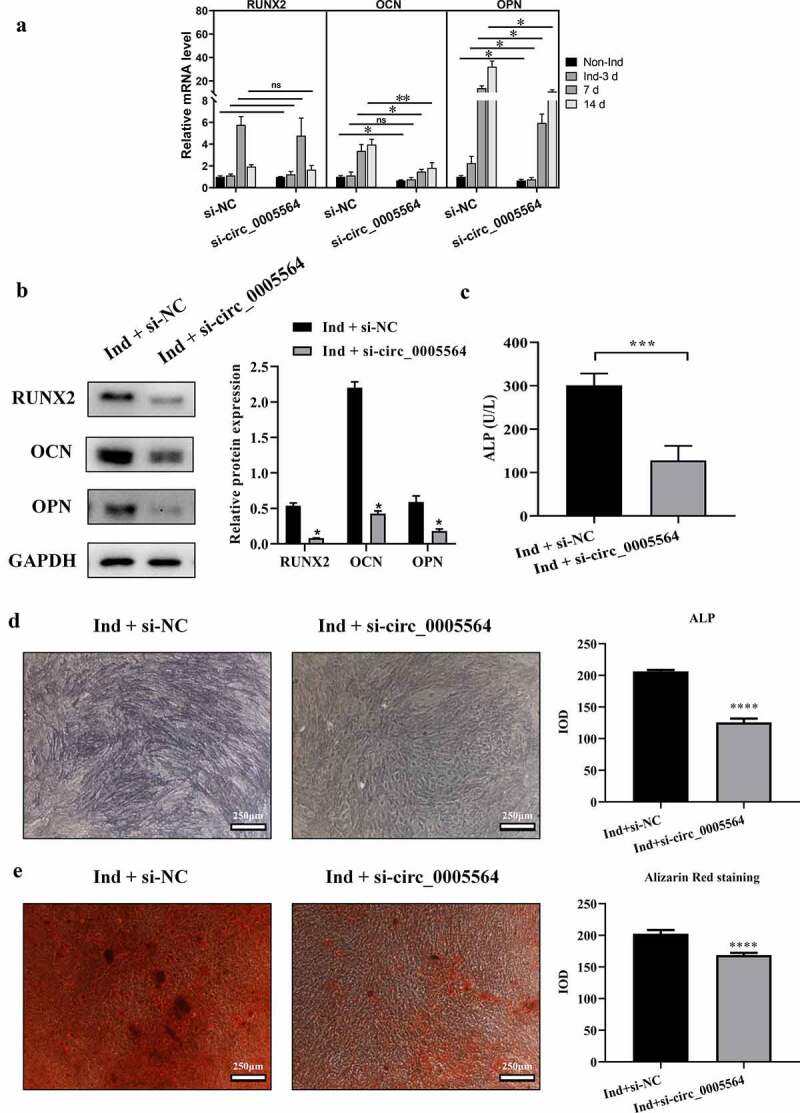
Circ_0005564 silencing inhibited BMSCs osteoblast differentiation. (a) qRT-PCR results of RUNX2, OCN, and OPN mRNA expression after circ_0005564 knockdown in BMSCs after induction at indicated time-points. *p < 0.05, **p < 0.01, N = 3. (b) The protein expression of RUNX2, OPN, and OCN were significantly decreased by circ_0005564 knockdown (*p < 0.05); (c) knockdown circ_0005564 significantly reduced ALP activity in BMSCs with osteogenic differentiation induction (***p < 0.001). (d) circ_0005564 knockdown reduced ALP protein expression in BMSCs with osteogenic differentiation induction. (e) alizarin red staining showed that circ_0005564 knockdown inhibited mineralized nodules formation in BMSCs with osteogenic differentiation induction

We further evaluated the effect of circ_0005564 on osteogenic differentiation. Circ_0005564 silencing inhibited the increased expression of OCN mRNA at the 7^th^ and 14^th^ day of induction, respectively (Figure 5(a)). It also reduced OPN mRNA expression at the 3^rd^, 7^th^, 14^th^ day of induction compared with the corresponding si-NC groups, respectively (Figure 5(a)). However, the RUNX2 mRNA expressions between silencing and negative control group at all three time-points after induction were comparable (Figure 5(a)). The protein expression level of osteogenic differentiation markers (RUNX2, OPN, and OCN) in BMSCs of circ_0005564 knockdown was significantly lower than that of the respective control group (p < 0.05) (Figure 5(b)). Moreover, circ_0005564 knockdown also reduced ALP activity (p < 0.001, Figure 5(b)), ALP expression (Figure 5(c)), and mineralized nodules (Figure 5(d)) in BMSCs with osteogenic differentiation.

### 3.6. Circ_0005564 did not affect FGFR1 gene expression

FGFR1 plays an essential role in osteoblast differentiation via regulating the balance between bone formation and bone remodeling [27]. As shown in Figure S1, circ_0005564 knockdown did not significantly change FGFR1 gene expression under control and osteogenic differentiation induction conditions.

### 3.7. Predicted potential targets of Circ_0005564

We used CircInteractome to predict the potential target of circ_0005564. The predicted binding miRNA of circ_0005564 was shown in the supplementary Table S2. Then, we adopted those miRNAs with a score ≥90 to predict their potential targets (shown in the supplementary Table S3). These results suggest that circ_0005564 might act as a ‘sponge’ to absorb a miRNA set and thus regulate its downstream gene expression.

## 4. Discussion

We profiled differentially expressed circRNAs using RNA-seq sequencing and verified 10 most significant differences of circRNAs expression in BMSCs of osteogenic differentiation. Among these 10 circRNAs, we performed the circRNA-miRNA-mRNA interaction network focused on 5 circRNAs (circ_005564, circ_0005255, circ_0006482, circ_0000707, and circ_0006083) with multiple targeted miRNAs. Eventually, our results revealed that circ_0005564 positively regulated osteogenic differentiation of BMSCs.

Osteogenic differentiation induction could activate the Ras/MAPK/Runx2 signaling pathway to promote the osteogenic differentiation of bone precursor cells, showing increased gene expression of ALP, OPN, and OCN [28]. An increase in ALP level and activity enhanced the calcification level of BMSCs and further increased the formation of mineralized nodules [29,30]. In our study, RUNX2, OPN, and OCN protein expression, ALP’s activity and protein expression, and mineralized nodules significantly increased in BMSCs treated with human mesenchymal stem cell osteogenic differentiation medium for 14 days, demonstrate that the osteogenic differentiation of BMSCs is successfully induced.

It has been reported that circRNAs were differentially expressed during the differentiation process of osteoblasts. CircRNA.5846, circRNA.19142, and circRNA.10042 are upregulated in differentiated MC3T3-E1 cells induced by bone morphogenetic protein 2 [31] (BMP2). Up-regulated circ CDR1as might act as a sponge for miR-7 to promote bone formation during the osteogenic differentiation of periodontal ligament stem cells [32]. CircRNAs including circ_0006618, circ_0005752, circRNA_0002890, and circ_0001421 were the most up-regulated genes in human adipose-derived stem cell (hADSC) osteogenesis [33]. In BMSCs, up-regulated circIGSF11 promoted osteogenic differentiation in BMSCs, while circCDR1 inhibited adipogenesis and osteogenic differentiation of BMSCs in femoral head necrosis caused by steroid hormones [34,35]. Circular RNA_0062582 targeted microRNA-145/core-binding factor subunit β (CBFB) axis to regulate osteogenic differentiation in human BMSCs [36]. These previous studies mainly revealed varied expression profiles of circRNAs during the osteogenic differentiation of different sources of stem cells, while our study uncovered the differentially expressed circRNAs profiles in BMSCs with osteogenic differentiation. Furthermore, in line with RNA sequencing results, we validated that circ_0109177, circ_0001085, circ_0005078, circ_0006083, circ_0006482, circ_0000707, circ_0005564 and circ_0005255 were up-regulated in BMSCs at the 14^th^ day of differentiation induction.

Hypoxia inhibited metabolic switch and mitochondrial function to inhibit the osteogenic differentiation, suggesting that metabolic conversion and mitochondrial activation are critical for MSCs osteogenic differentiation [37]. Down-regulated hypoxia-inducible factor 1 (HIF-1) is related to mitochondrial phosphorylation activation during differentiation [38]. In our study, the bioinformatic analysis indicated that the potential biological function of the differentially expressed circRNAs is mainly related to metabolic pathway, and they enriched in osteoblast differentiation BMSCs. These results suggest that the differentially expressed circRNAs participate in the regulation of osteogenic differentiation-related pathways.

CircRNAs play a regulatory role in osteogenic differentiation through miRNA adsorption: silencing circIGSF11 promoted osteogenic differentiation and increased miR-199b-5p expression; circCDR1as-miR-7-5p-WNT5B pathway inhibited bone differentiation; and fat-derived circPOMT1 and circMCM3AP targeted miR-6881-3p to regulate osteogenic differentiation [34,35,39]. Previous studies have found that circDAB1 promoted osteogenic differentiation by increasing miR-1270 and miR-944 expression, and the circRUNX2-miR-203 axis promoted osteogenic differentiation by reducing RUNX2 expression [11,40]. Our study further outlined the circRNA-miRNA-mRNA networks of five differentially expressed circRNAs with the most up-regulation after 14 days of BMSC induction, including circ_0005564, circ_0005255, circ_0006482, circ_0000707, and circ_0006083, providing clues for subsequent functional investigation. However, out of these five circRNAs, we only found that circ_0005564 knockdown significantly inhibited the expression of osteogenic differentiation markers (RUNX2, OCN, and OPN). Our results showed that circ_0005564 knockdown significantly reduced ALP protein expression and activity, and inhibited mineralized nodules formation, suggesting circ_0005564 plays a key role in BMSCs osteogenic differentiation. Moreover, the bioinformatic analysis indicates that circ_0005564 might regulate the downstream targets through miRNA–mRNA interaction, which requires future investigation. Although our study demonstrates that circ_0005564 is related to osteogenic differentiation of BMSCs, future in vivo and in vitro studies and the underlying molecular mechanism of circ_0005564 are in need.

## 5. Conclusion

In summary, our study uncovered the differentially expressed circRNAs profile in BMSCs of osteogenic differentiation. This study demonstrates that circ_0005564 might act as a positive regulator of osteogenic differentiation in BMSCs, providing a potential target for the treatment of OP.

## Supplementary Material

Supplemental Material

## Data Availability

All data generated or analyzed during this study are included in this published article.
