# Supplementary material for: Circular RNA Circ_0005564 promotes osteogenic differentiation of bone marrow mesenchymal cells in osteoporosis
Source: Bioengineered. 2021 Aug 10;12(1):4911–23. doi: 10.1080/21655979.2021.1959865 (PMC8806437; doi:10.1080/21655979.2021.1959865)
Supplement: Supplemental Material [file KBIE_A_1959865_SM0129.zip › suppl/Table S1 new.docx]

Table S1. List of osteogenic differentiation related pathways.

| NAME | PROBE | DESCRIPTION <br> (from dataset) | GENE SYMBOL | GENE_TITLE | RANK IN GENE LIST | RANK METRIC SCORE | RUNNING ES | CORE ENRICHMENT |
| --- | --- | --- | --- | --- | --- | --- | --- | --- |
| row_0 | NTRK2 | na | NTRK2 | neurotrophic tyrosine kinase, receptor, type 2 | 106 | 4.641681 | -0.01101 | Yes |
| row_1 | NGF | na | null | null | 134 | 4.575177 | -0.00651 | Yes |
| row_2 | FZD7 | na | FZD7 | frizzled homolog 7 (Drosophila) | 182 | 4.47188 | -0.0062 | Yes |
| row_3 | APC2 | na | APC2 | adenomatosis polyposis coli 2 | 236 | 4.340792 | -0.00736 | Yes |
| row_4 | TGFBR1 | na | TGFBR1 | transforming growth factor, beta receptor I (activin A receptor type II-like kinase, 53kDa) | 239 | 4.337851 | 0.00158 | Yes |
| row_5 | FGF11 | na | FGF11 | fibroblast growth factor 11 | 249 | 4.317498 | 0.009088 | Yes |
| row_6 | NKD2 | na | NKD2 | naked cuticle homolog 2 (Drosophila) | 256 | 4.295444 | 0.017143 | Yes |
| row_7 | MAPT | na | MAPT | microtubule-associated protein tau | 289 | 4.185752 | 0.019811 | Yes |
| row_8 | TCF7L1 | na | TCF7L1 | transcription factor 7-like 1 (T-cell specific, HMG-box) | 312 | 4.120344 | 0.02432 | Yes |
| row_9 | GLI2 | na | GLI2 | GLI-Kruppel family member GLI2 | 321 | 4.102984 | 0.031565 | Yes |
| row_10 | FZD3 | na | FZD3 | frizzled homolog 3 (Drosophila) | 354 | 4.019793 | 0.033876 | Yes |
| row_11 | CCND2 | na | CCND2 | cyclin D2 | 382 | 3.97558 | 0.037082 | Yes |
| row_12 | SMO | na | SMO | smoothened homolog (Drosophila) | 407 | 3.93592 | 0.040798 | Yes |
| row_13 | DUSP8 | na | DUSP8 | dual specificity phosphatase 8 | 410 | 3.929145 | 0.048857 | Yes |
| row_14 | DTX4 | na | DTX4 | deltex 4 homolog (Drosophila) | 420 | 3.910062 | 0.055488 | Yes |
| row_15 | TGFB2 | na | TGFB2 | transforming growth factor, beta 2 | 426 | 3.883694 | 0.062855 | Yes |
| row_16 | RPS6KA6 | na | RPS6KA6 | ribosomal protein S6 kinase, 90kDa, polypeptide 6 | 464 | 3.792713 | 0.063687 | Yes |
| row_17 | CACNA1A | na | CACNA1A | calcium channel, voltage-dependent, P/Q type, alpha 1A subunit | 465 | 3.789154 | 0.071841 | Yes |
| row_18 | PORCN | na | PORCN | porcupine homolog (Drosophila) | 489 | 3.730207 | 0.075312 | Yes |
| row_19 | PLCB1 | na | PLCB1 | phospholipase C, beta 1 (phosphoinositide-specific) | 514 | 3.68888 | 0.078496 | Yes |
| row_20 | BCL2 | na | BCL2 | B-cell CLL/lymphoma 2 | 525 | 3.673717 | 0.084421 | Yes |
| row_21 | FGF2 | na | FGF2 | fibroblast growth factor 2 (basic) | 549 | 3.622977 | 0.087661 | Yes |
| row_22 | EGFR | na | EGFR | epidermal growth factor receptor (erythroblastic leukemia viral (v-erb-b) oncogene homolog, avian) | 594 | 3.524328 | 0.086529 | Yes |
| row_23 | DKK1 | na | DKK1 | dickkopf homolog 1 (Xenopus laevis) | 620 | 3.491753 | 0.08909 | Yes |
| row_24 | NOTCH1 | na | NOTCH1 | Notch homolog 1, translocation-associated (Drosophila) | 633 | 3.473348 | 0.094188 | Yes |
| row_25 | FGF5 | na | FGF5 | fibroblast growth factor 5 | 642 | 3.459754 | 0.100048 | Yes |
| row_26 | JAG2 | na | JAG2 | jagged 2 | 648 | 3.449586 | 0.106481 | Yes |
| row_27 | PLCB4 | na | PLCB4 | phospholipase C, beta 4 | 650 | 3.449214 | 0.113705 | Yes |
| row_28 | RASGRF2 | na | RASGRF2 | Ras protein-specific guanine nucleotide-releasing factor 2 | 691 | 3.381195 | 0.113057 | Yes |
| row_29 | MECOM | na | null | null | 707 | 3.356752 | 0.117309 | Yes |
| row_30 | CACNG7 | na | CACNG7 | calcium channel, voltage-dependent, gamma subunit 7 | 776 | 3.262329 | 0.110859 | Yes |
| row_31 | WNT5A | na | WNT5A | wingless-type MMTV integration site family, member 5A | 778 | 3.260582 | 0.117678 | Yes |
| row_32 | NTF3 | na | NTF3 | neurotrophin 3 | 836 | 3.197116 | 0.113266 | Yes |
| row_33 | MMP7 | na | MMP7 | matrix metallopeptidase 7 (matrilysin, uterine) | 854 | 3.178158 | 0.116737 | Yes |
| row_34 | BDNF | na | BDNF | brain-derived neurotrophic factor | 874 | 3.158929 | 0.119771 | Yes |
| row_35 | PRICKLE1 | na | PRICKLE1 | prickle homolog 1 (Drosophila) | 913 | 3.126013 | 0.118971 | Yes |
| row_36 | DDIT3 | na | DDIT3 | DNA-damage-inducible transcript 3 | 914 | 3.126013 | 0.125698 | Yes |
| row_37 | DUSP10 | na | DUSP10 | dual specificity phosphatase 10 | 925 | 3.114158 | 0.130418 | Yes |
| row_38 | FZD8 | na | FZD8 | frizzled homolog 8 (Drosophila) | 976 | 3.059816 | 0.127098 | Yes |
| row_39 | TRAF6 | na | TRAF6 | TNF receptor-associated factor 6 | 1016 | 3.023955 | 0.125879 | Yes |
| row_40 | CACNB1 | na | CACNB1 | calcium channel, voltage-dependent, beta 1 subunit | 1044 | 2.994075 | 0.126974 | Yes |
| row_41 | PRKACB | na | PRKACB | protein kinase, cAMP-dependent, catalytic, beta | 1057 | 2.976024 | 0.131001 | Yes |
| row_42 | MAP3K1 | na | MAP3K1 | mitogen-activated protein kinase kinase kinase 1 | 1112 | 2.926575 | 0.126601 | Yes |
| row_43 | AXIN2 | na | AXIN2 | axin 2 (conductin, axil) | 1200 | 2.846968 | 0.115493 | Yes |
| row_44 | TAOK1 | na | TAOK1 | TAO kinase 1 | 1238 | 2.814379 | 0.11422 | Yes |
| row_45 | APC | na | APC | adenomatosis polyposis coli | 1280 | 2.783368 | 0.112088 | Yes |
| row_46 | SPOPL | na | null | null | 1281 | 2.782196 | 0.118075 | Yes |
| row_47 | PTCH1 | na | PTCH1 | patched homolog 1 (Drosophila) | 1287 | 2.776962 | 0.12306 | Yes |
| row_48 | CSNK1G3 | na | CSNK1G3 | casein kinase 1, gamma 3 | 1292 | 2.771937 | 0.128233 | Yes |
| row_49 | FGF7 | na | FGF7 | fibroblast growth factor 7 (keratinocyte growth factor) | 1297 | 2.766031 | 0.133393 | Yes |
| row_50 | FZD6 | na | FZD6 | frizzled homolog 6 (Drosophila) | 1315 | 2.743781 | 0.135929 | Yes |
| row_51 | LRP5 | na | LRP5 | low density lipoprotein receptor-related protein 5 | 1388 | 2.696882 | 0.12747 | Yes |
| row_52 | CREBBP | na | CREBBP | CREB binding protein (Rubinstein-Taybi syndrome) | 1403 | 2.686827 | 0.130478 | Yes |
| row_53 | EP300 | na | EP300 | E1A binding protein p300 | 1425 | 2.661541 | 0.132046 | Yes |
| row_54 | RBPJ | na | null | null | 1471 | 2.635783 | 0.128803 | Yes |
| row_55 | CCND1 | na | CCND1 | cyclin D1 | 1481 | 2.629575 | 0.132679 | Yes |
| row_56 | GADD45A | na | GADD45A | growth arrest and DNA-damage-inducible, alpha | 1491 | 2.619431 | 0.136533 | Yes |
| row_57 | GSK3B | na | GSK3B | glycogen synthase kinase 3 beta | 1572 | 2.563496 | 0.126202 | No |
| row_58 | PPM1A | na | PPM1A | protein phosphatase 1A (formerly 2C), magnesium-dependent, alpha isoform | 1686 | 2.497456 | 0.109191 | No |
| row_59 | JUN | na | JUN | jun oncogene | 1744 | 2.46245 | 0.103198 | No |
| row_60 | ROCK2 | na | ROCK2 | Rho-associated, coiled-coil containing protein kinase 2 | 1749 | 2.458918 | 0.107697 | No |
| row_61 | MAML1 | na | MAML1 | mastermind-like 1 (Drosophila) | 1751 | 2.456737 | 0.112786 | No |
| row_62 | SMURF2 | na | SMURF2 | SMAD specific E3 ubiquitin protein ligase 2 | 1781 | 2.441415 | 0.112295 | No |
| row_63 | NOTCH2 | na | NOTCH2 | Notch homolog 2 (Drosophila) | 1790 | 2.435236 | 0.115951 | No |
| row_64 | FZD5 | na | FZD5 | frizzled homolog 5 (Drosophila) | 1829 | 2.414471 | 0.113619 | No |
| row_65 | RASA2 | na | RASA2 | RAS p21 protein activator 2 | 1895 | 2.379587 | 0.105863 | No |
| row_66 | SIAH1 | na | SIAH1 | seven in absentia homolog 1 (Drosophila) | 1901 | 2.376348 | 0.109986 | No |
| row_67 | TCF7L2 | na | TCF7L2 | transcription factor 7-like 2 (T-cell specific, HMG-box) | 1920 | 2.3652 | 0.11151 | No |
| row_68 | CRKL | na | CRKL | v-crk sarcoma virus CT10 oncogene homolog (avian)-like | 1935 | 2.354759 | 0.113804 | No |
| row_69 | GNG12 | na | GNG12 | guanine nucleotide binding protein (G protein), gamma 12 | 1942 | 2.353565 | 0.11768 | No |
| row_70 | NFATC4 | na | NFATC4 | nuclear factor of activated T-cells, cytoplasmic, calcineurin-dependent 4 | 1967 | 2.341591 | 0.117965 | No |
| row_71 | PSEN2 | na | PSEN2 | presenilin 2 (Alzheimer disease 4) | 1971 | 2.337042 | 0.1224 | No |
| row_72 | TBL1X | na | TBL1X | transducin (beta)-like 1X-linked | 2023 | 2.313745 | 0.117276 | No |
| row_73 | STK4 | na | STK4 | serine/threonine kinase 4 | 2024 | 2.313319 | 0.122254 | No |
| row_74 | MAPK8 | na | MAPK8 | mitogen-activated protein kinase 8 | 2101 | 2.268287 | 0.11208 | No |
| row_75 | CHUK | na | CHUK | conserved helix-loop-helix ubiquitous kinase | 2124 | 2.254821 | 0.112574 | No |
| row_76 | BRAF | na | BRAF | v-raf murine sarcoma viral oncogene homolog B1 | 2202 | 2.222368 | 0.102103 | No |
| row_77 | FBXW11 | na | FBXW11 | F-box and WD-40 domain protein 11 | 2222 | 2.212926 | 0.103101 | No |
| row_78 | CDON | na | CDON | Cdon homolog (mouse) | 2228 | 2.210612 | 0.106868 | No |
| row_79 | RAPGEF2 | na | RAPGEF2 | Rap guanine nucleotide exchange factor (GEF) 2 | 2232 | 2.207895 | 0.111024 | No |
| row_80 | NFATC3 | na | NFATC3 | nuclear factor of activated T-cells, cytoplasmic, calcineurin-dependent 3 | 2326 | 2.16117 | 0.097252 | No |
| row_81 | MAP3K2 | na | MAP3K2 | mitogen-activated protein kinase kinase kinase 2 | 2342 | 2.152015 | 0.098912 | No |
| row_82 | PPM1B | na | PPM1B | protein phosphatase 1B (formerly 2C), magnesium-dependent, beta isoform | 2343 | 2.151482 | 0.103541 | No |
| row_83 | SOS2 | na | SOS2 | son of sevenless homolog 2 (Drosophila) | 2401 | 2.120342 | 0.096813 | No |
| row_84 | MAPK14 | na | MAPK14 | mitogen-activated protein kinase 14 | 2418 | 2.112882 | 0.09819 | No |
| row_85 | CSNK2A1 | na | CSNK2A1 | casein kinase 2, alpha 1 polypeptide | 2434 | 2.104913 | 0.099748 | No |
| row_86 | ELK4 | na | ELK4 | ELK4, ETS-domain protein (SRF accessory protein 1) | 2472 | 2.087117 | 0.09691 | No |
| row_87 | SFRP4 | na | SFRP4 | secreted frizzled-related protein 4 | 2491 | 2.080463 | 0.097821 | No |
| row_88 | CACNA1C | na | CACNA1C | calcium channel, voltage-dependent, L type, alpha 1C subunit | 2568 | 2.052753 | 0.087183 | No |
| row_89 | SMAD4 | na | SMAD4 | SMAD, mothers against DPP homolog 4 (Drosophila) | 2582 | 2.046607 | 0.089012 | No |
| row_90 | MAPK1 | na | MAPK1 | mitogen-activated protein kinase 1 | 2621 | 2.030832 | 0.085854 | No |
| row_91 | MAP2K1 | na | MAP2K1 | mitogen-activated protein kinase kinase 1 | 2748 | 1.975178 | 0.065144 | No |
| row_92 | RASA1 | na | RASA1 | RAS p21 protein activator (GTPase activating protein) 1 | 2775 | 1.964154 | 0.064221 | No |
| row_93 | AKT3 | na | AKT3 | v-akt murine thymoma viral oncogene homolog 3 (protein kinase B, gamma) | 2788 | 1.958505 | 0.066058 | No |
| row_94 | CSNK1A1 | na | CSNK1A1 | casein kinase 1, alpha 1 | 2849 | 1.931847 | 0.058329 | No |
| row_95 | CRK | na | CRK | v-crk sarcoma virus CT10 oncogene homolog (avian) | 2851 | 1.931396 | 0.062287 | No |
| row_96 | FGFR2 | na | FGFR2 | ffer syndrome, Jackson-Weiss syndrome) | 2869 | 1.925302 | 0.063063 | No |
| row_97 | BTRC | na | BTRC | beta-transducin repeat containing | 2877 | 1.921258 | 0.065811 | No |
| row_98 | CSNK1G1 | na | CSNK1G1 | casein kinase 1, gamma 1 | 2898 | 1.913489 | 0.065966 | No |
| row_99 | CTNNB1 | na | CTNNB1 | catenin (cadherin-associated protein), beta 1, 88kDa | 3019 | 1.86293 | 0.046204 | No |
| row_100 | DUSP3 | na | DUSP3 | dual specificity phosphatase 3 (vaccinia virus phosphatase VH1-related) | 3045 | 1.852988 | 0.045239 | No |
| row_101 | NF1 | na | NF1 | neurofibromin 1 (neurofibromatosis, von Recklinghausen disease, Watson disease) | 3066 | 1.846979 | 0.045251 | No |
| row_102 | FGFR1 | na | FGFR1 | fibroblast growth factor receptor 1 (fms-related tyrosine kinase 2, Pfeiffer syndrome) | 3114 | 1.830519 | 0.03988 | No |
| row_103 | KRAS | na | KRAS | v-Ki-ras2 Kirsten rat sarcoma viral oncogene homolog | 3129 | 1.825353 | 0.041034 | No |
| row_104 | LRP6 | na | LRP6 | low density lipoprotein receptor-related protein 6 | 3156 | 1.816022 | 0.039792 | No |
| row_105 | WNT5B | na | WNT5B | wingless-type MMTV integration site family, member 5B | 3268 | 1.769878 | 0.021612 | No |
| row_106 | LAMTOR3 | na | null | null | 3316 | 1.753121 | 0.016074 | No |
| row_107 | SENP2 | na | SENP2 | SUMO1/sentrin/SMT3 specific peptidase 2 | 3342 | 1.742277 | 0.01487 | No |
| row_108 | ATF2 | na | ATF2 | activating transcription factor 2 | 3365 | 1.734468 | 0.014245 | No |
| row_109 | RELA | na | RELA | v-rel reticuloendotheliosis viral oncogene homolog A, nuclear factor of kappa light polypeptide gene enhancer in B-cells 3, p65 (avian) | 3383 | 1.726398 | 0.014592 | No |
| row_110 | ATF4 | na | ATF4 | activating transcription factor 4 (tax-responsive enhancer element B67) | 3421 | 1.711508 | 0.010946 | No |
| row_111 | PAK2 | na | PAK2 | p21 (CDKN1A)-activated kinase 2 | 3429 | 1.707516 | 0.013233 | No |
| row_112 | KIF3A | na | KIF3A | kinesin family member 3A | 3444 | 1.699895 | 0.014118 | No |
| row_113 | MAP2K7 | na | MAP2K7 | mitogen-activated protein kinase kinase 7 | 3483 | 1.684322 | 0.010215 | No |
| row_114 | VANGL2 | na | VANGL2 | vang-like 2 (van gogh, Drosophila) | 3606 | 0.582749 | -0.0127 | No |
| row_115 | CACNA1H | na | CACNA1H | calcium channel, voltage-dependent, alpha 1H subunit | 3621 | 0.335353 | -0.01475 | No |
| row_116 | CACNG8 | na | CACNG8 | calcium channel, voltage-dependent, gamma subunit 8 | 3622 | 0.311529 | -0.01408 | No |
| row_117 | FGF12 | na | FGF12 | fibroblast growth factor 12 | 3640 | -0.51653 | -0.01634 | No |
| row_118 | HES5 | na | HES5 | hairy and enhancer of split 5 (Drosophila) | 3678 | -1.49842 | -0.02044 | No |
| row_119 | TGFB1 | na | TGFB1 | transforming growth factor, beta 1 (Camurati-Engelmann disease) | 3740 | -1.72091 | -0.02882 | No |
| row_120 | JUND | na | JUND | jun D proto-oncogene | 3744 | -1.7299 | -0.02569 | No |
| row_121 | NFKB1 | na | NFKB1 | nuclear factor of kappa light polypeptide gene enhancer in B-cells 1 (p105) | 3768 | -1.75374 | -0.02648 | No |
| row_122 | PLA2G4C | na | PLA2G4C | phospholipase A2, group IVC (cytosolic, calcium-independent) | 3776 | -1.76581 | -0.02406 | No |
| row_123 | SMAD3 | na | SMAD3 | SMAD, mothers against DPP homolog 3 (Drosophila) | 3835 | -1.8253 | -0.03162 | No |
| row_124 | PDGFRB | na | PDGFRB | platelet-derived growth factor receptor, beta polypeptide | 3875 | -1.88066 | -0.0353 | No |
| row_125 | DAAM2 | na | DAAM2 | dishevelled associated activator of morphogenesis 2 | 3876 | -1.8828 | -0.03125 | No |
| row_126 | NFKB2 | na | NFKB2 | nuclear factor of kappa light polypeptide gene enhancer in B-cells 2 (p49/p100) | 3894 | -1.89953 | -0.03053 | No |
| row_127 | ARRB1 | na | ARRB1 | arrestin, beta 1 | 3934 | -1.94426 | -0.03407 | No |
| row_128 | HHIP | na | HHIP | hedgehog interacting protein | 4072 | -2.1414 | -0.05661 | No |
| row_129 | PDGFRA | na | PDGFRA | platelet-derived growth factor receptor, alpha polypeptide | 4119 | -2.20602 | -0.06097 | No |
| row_130 | DUSP1 | na | DUSP1 | dual specificity phosphatase 1 | 4205 | -2.36857 | -0.07271 | No |
| row_131 | SHH | na | SHH | sonic hedgehog homolog (Drosophila) | 4209 | -2.37209 | -0.0682 | No |
| row_132 | RAC2 | na | RAC2 | ras-related C3 botulinum toxin substrate 2 (rho family, small GTP binding protein Rac2) | 4253 | -2.44315 | -0.07146 | No |
| row_133 | MAP2K3 | na | MAP2K3 | mitogen-activated protein kinase kinase 3 | 4323 | -2.56679 | -0.07961 | No |
| row_134 | RELB | na | RELB | v-rel reticuloendotheliosis viral oncogene homolog B, nuclear factor of kappa light polypeptide gene enhancer in B-cells 3 (avian) | 4339 | -2.59444 | -0.077 | No |
| row_135 | IL1R1 | na | IL1R1 | interleukin 1 receptor, type I | 4421 | -2.77902 | -0.08706 | No |
| row_136 | ADRBK2 | na | ADRBK2 | adrenergic, beta, receptor kinase 2 | 4425 | -2.78724 | -0.08166 | No |
| row_137 | LEF1 | na | LEF1 | lymphoid enhancer-binding factor 1 | 4512 | -2.9724 | -0.0923 | No |
| row_138 | PLA2G4A | na | PLA2G4A | phospholipase A2, group IVA (cytosolic, calcium-dependent) | 4515 | -2.98065 | -0.08628 | No |
| row_139 | WNT2B | na | WNT2B | wingless-type MMTV integration site family, member 2B | 4565 | -3.11567 | -0.08928 | No |
| row_140 | CD14 | na | CD14 | CD14 molecule | 4593 | -3.15887 | -0.08783 | No |
| row_141 | NFATC2 | na | NFATC2 | nuclear factor of activated T-cells, cytoplasmic, calcineurin-dependent 2 | 4626 | -3.23632 | -0.08721 | No |
| row_142 | SERPINF1 | na | SERPINF1 | serpin peptidase inhibitor, clade F (alpha-2 antiplasmin, pigment epithelium derived factor), member 1 | 4730 | -3.53109 | -0.10001 | No |
| row_143 | DUSP4 | na | DUSP4 | dual specificity phosphatase 4 | 4758 | -3.61483 | -0.09758 | No |
| row_144 | MAPK13 | na | MAPK13 | mitogen-activated protein kinase 13 | 4769 | -3.64319 | -0.09172 | No |
| row_145 | CACNA2D4 | na | CACNA2D4 | calcium channel, voltage-dependent, alpha 2/delta subunit 4 | 4781 | -3.67094 | -0.086 | No |
| row_146 | SFRP5 | na | SFRP5 | secreted frizzled-related protein 5 | 4860 | -3.92922 | -0.093 | No |
| row_147 | MAP3K14 | na | MAP3K14 | mitogen-activated protein kinase kinase kinase 14 | 4878 | -3.96087 | -0.08784 | No |
| row_148 | FOS | na | FOS | v-fos FBJ murine osteosarcoma viral oncogene homolog | 4880 | -3.96271 | -0.07951 | No |
| row_149 | CACNB2 | na | CACNB2 | calcium channel, voltage-dependent, beta 2 subunit | 4891 | -4.00077 | -0.07289 | No |
| row_150 | TGFB3 | na | TGFB3 | transforming growth factor, beta 3 | 4892 | -4.00821 | -0.06426 | No |
| row_151 | CACNA1G | na | CACNA1G | calcium channel, voltage-dependent, alpha 1G subunit | 4903 | -4.04184 | -0.05754 | No |
| row_152 | WNT16 | na | WNT16 | wingless-type MMTV integration site family, member 16 | 4923 | -4.08584 | -0.05252 | No |
| row_153 | IL1R2 | na | IL1R2 | interleukin 1 receptor, type II | 4937 | -4.12263 | -0.04622 | No |
| row_154 | DUSP5 | na | DUSP5 | dual specificity phosphatase 5 | 4956 | -4.17884 | -0.04079 | No |
| row_155 | MAP3K8 | na | MAP3K8 | mitogen-activated protein kinase kinase kinase 8 | 4966 | -4.20767 | -0.03352 | No |
| row_156 | DUSP2 | na | DUSP2 | dual specificity phosphatase 2 | 5013 | -4.36651 | -0.03324 | No |
| row_157 | MAP2K6 | na | MAP2K6 | mitogen-activated protein kinase kinase 6 | 5031 | -4.42036 | -0.02709 | No |
| row_158 | IL1A | na | IL1A | interleukin 1, alpha | 5057 | -4.51391 | -0.02233 | No |
| row_159 | MAPK10 | na | MAPK10 | mitogen-activated protein kinase 10 | 5129 | -4.72584 | -0.02623 | No |
| row_160 | IL1B | na | IL1B | interleukin 1, beta | 5144 | -4.76431 | -0.01875 | No |
| row_161 | HES1 | na | HES1 | hairy and enhancer of split 1, (Drosophila) | 5154 | -4.79593 | -0.01021 | No |
| row_162 | RASGRP2 | na | RASGRP2 | RAS guanyl releasing protein 2 (calcium and DAG-regulated) | 5185 | -4.89495 | -0.00562 | No |
| row_163 | NR4A1 | na | NR4A1 | nuclear receptor subfamily 4, group A, member 1 | 5191 | -4.9121 | 0.003962 | No |
