# Supplementary material for: Circular RNA Circ_0005564 promotes osteogenic differentiation of bone marrow mesenchymal cells in osteoporosis
Source: Bioengineered. 2021 Aug 10;12(1):4911–23. doi: 10.1080/21655979.2021.1959865 (PMC8806437; doi:10.1080/21655979.2021.1959865)
Supplement: Supplemental Material [file KBIE_A_1959865_SM0129.zip › suppl/Table S2 new.docx]

| **Table S2.TargetScan miRNA predictions of hsa_circ_0005564** | | | | | | | | | | | |
| --- | --- | --- | --- | --- | --- | --- | --- | --- | --- | --- | --- |
| **CircRNA  Mirbase ID** | **CircRNA (Top) - miRNA (Bottom) pairing** | **Site Type** | **CircRNA Start** | **CircRNA End** | **3' pairing** | **local AU** | **position** | **TA** | **SPS** | **context+ score** | **context+ score percentile** |
| [hsa_circ_0005564](http://www.circbase.org/cgi-bin/singlerecord.cgi?id=hsa_circ_0005564) (5' ... 3')  [hsa-miR-1208](http://www.mirbase.org/cgi-bin/mirna_entry.pl?acc=hsa-miR-1208) (3' ... 5') | GUUGCCCGCCAACAA-AACAGUGG      \|\|\|\|\|      \|\|\|\|\|\|\|      AGGCGGACAGACUUGUCACU | 7mer-m8 | 174 | 180 | -0.025 | 0.046 | -0.043 | 0.011 | 0.020 | -0.111 | **90** |
| [hsa_circ_0005564](http://www.circbase.org/cgi-bin/singlerecord.cgi?id=hsa_circ_0005564) (5' ... 3')  [hsa-miR-1208](http://www.mirbase.org/cgi-bin/mirna_entry.pl?acc=hsa-miR-1208) (3' ... 5') | UCAUGUGUAAGGUGUACAGUGAC                 \|\|\|\|\|\|      AGGCGGACAGACUUGUCACU | 7mer-1a | 218 | 224 | 0.001 | 0.057 | -0.040 | 0.011 | -0.013 | -0.058 | 78 |
| [hsa_circ_0005564](http://www.circbase.org/cgi-bin/singlerecord.cgi?id=hsa_circ_0005564) (5' ... 3')  [hsa-miR-1231](http://www.mirbase.org/cgi-bin/mirna_entry.pl?acc=hsa-miR-1231) (3' ... 5') | GGGAGCAAGAUUGGCCCAGACAA                \|\|\|\|\|\|\|     CGUCGACAGGCGGGUCUGUG | 8mer-1a | 283 | 290 | 0.003 | 0.101 | -0.108 | -0.001 | -0.063 | -0.315 | **97** |
| [hsa_circ_0005564](http://www.circbase.org/cgi-bin/singlerecord.cgi?id=hsa_circ_0005564) (5' ... 3')  [hsa-miR-1243](http://www.mirbase.org/cgi-bin/mirna_entry.pl?acc=hsa-miR-1243) (3' ... 5') | GACCCGCAGCCGCACAUCCAGUG                \|\|\|\|\|\|\|   GUGAGGAUAUUAACUAGGUCAA | 7mer-m8 | 238 | 244 | 0.021 | 0.091 | -0.052 | -0.012 | -0.002 | -0.074 | 68 |
| [hsa_circ_0005564](http://www.circbase.org/cgi-bin/singlerecord.cgi?id=hsa_circ_0005564) (5' ... 3')  [hsa-miR-1272](http://www.mirbase.org/cgi-bin/mirna_entry.pl?acc=hsa-miR-1272) (3' ... 5') | UUAUGCCACCUGGAGCAUCAUAA                    \|\|\|\|\|\|   AAAGUCUUAAACGACGGUAGUAGUAG | 7mer-1a | 21 | 27 | -0.002 | 0.034 | -0.048 | -0.006 | 0.033 | -0.063 | 82 |
| [hsa_circ_0005564](http://www.circbase.org/cgi-bin/singlerecord.cgi?id=hsa_circ_0005564) (5' ... 3')  [hsa-miR-1286](http://www.mirbase.org/cgi-bin/mirna_entry.pl?acc=hsa-miR-1286) (3' ... 5') | CCCCUCACCGGCCCAUCCUGCAA     \|\|\|\|        \|\|\|\|\|\|   UCCCGAGUAGAACC--AGGACGU | 7mer-1a | 146 | 152 | -0.009 | 0.068 | -0.034 | 0.009 | -0.069 | -0.109 | 72 |
| [hsa_circ_0005564](http://www.circbase.org/cgi-bin/singlerecord.cgi?id=hsa_circ_0005564) (5' ... 3')  [hsa-miR-1322](http://www.mirbase.org/cgi-bin/mirna_entry.pl?acc=hsa-miR-1322) (3' ... 5') | GUUAUGCCACCUGGAGCAUCAUA                \|\|\|\|\|\|\|      GUCGUAGUCGUCGUAGUAG | 8mer-1a | 20 | 27 | 0.003 | 0.035 | -0.109 | -0.025 | 0.009 | -0.334 | **99** |
| [hsa_circ_0005564](http://www.circbase.org/cgi-bin/singlerecord.cgi?id=hsa_circ_0005564) (5' ... 3')  [hsa-miR-140-3p](http://www.mirbase.org/cgi-bin/mirna_entry.pl?acc=hsa-miR-140-3p) (3' ... 5') | GCAUCAUAAUGGACUCUGUGGUG                \|\|\|\|\|\|\|    GGCACCAAGAUGGGACACCAU | 7mer-m8 | 35 | 41 | 0.021 | 0.037 | -0.057 | 0.009 | -0.032 | -0.142 | 89 |
| [hsa_circ_0005564](http://www.circbase.org/cgi-bin/singlerecord.cgi?id=hsa_circ_0005564) (5' ... 3')  [hsa-miR-147b](http://www.mirbase.org/cgi-bin/mirna_entry.pl?acc=hsa-miR-147b) (3' ... 5') | UACAGUGACCCGCAGCCGCACAU                  \|\|\|\|\|\|\|     AUCGUCUUCGUAAAGGCGUGUG | 7mer-m8 | 232 | 238 | -0.007 | 0.063 | -0.051 | -0.081 | -0.064 | -0.26 | 86 |
| [hsa_circ_0005564](http://www.circbase.org/cgi-bin/singlerecord.cgi?id=hsa_circ_0005564) (5' ... 3')  [hsa-miR-191](http://www.mirbase.org/cgi-bin/mirna_entry.pl?acc=hsa-miR-191) (3' ... 5') | NNNNNNNNNNNNNNNGUCCGUUAU                 \|\|\|\|\|\|   GUCGACGAAAACCCUAAGGCAAC | 7mer-1a | 2 | 8 | too_close | too_close | too_close | too_close | too_close | too_close | NA |
| [hsa_circ_0005564](http://www.circbase.org/cgi-bin/singlerecord.cgi?id=hsa_circ_0005564) (5' ... 3')  [hsa-miR-515-5p](http://www.mirbase.org/cgi-bin/mirna_entry.pl?acc=hsa-miR-515-5p) (3' ... 5') | ACUACACCUGCAUUGUGGAGAAU                  \|\|\|\|\|\|   GUCUUUCACGAAAGAAAACCUCUU | 7mer-1a | 74 | 80 | 0.001 | 0.007 | -0.042 | 0.023 | -0.034 | -0.119 | 85 |
| [hsa_circ_0005564](http://www.circbase.org/cgi-bin/singlerecord.cgi?id=hsa_circ_0005564) (5' ... 3')  [hsa-miR-604](http://www.mirbase.org/cgi-bin/mirna_entry.pl?acc=hsa-miR-604) (3' ... 5') | AGGUGUACAGUGACC--CGCAGCCG           \|\|\|    \|\|\|\|\|\|\|        CAGGACUUAAGGCGUCGGA | 7mer-m8 | 227 | 233 | -0.016 | 0.071 | -0.050 | -0.003 | -0.099 | -0.217 | **92** |
| [hsa_circ_0005564](http://www.circbase.org/cgi-bin/singlerecord.cgi?id=hsa_circ_0005564) (5' ... 3')  [hsa-miR-636](http://www.mirbase.org/cgi-bin/mirna_entry.pl?acc=hsa-miR-636) (3' ... 5') | CACAUCCAGUGGCUAAAGCACAU                   \|\|\|\|\|\|     ACGCCCGCCCUGCUCGUUCGUGU | 7mer-1a | 250 | 256 | 0.004 | 0.036 | -0.044 | 0.001 | -0.013 | -0.09 | 78 |
| [hsa_circ_0005564](http://www.circbase.org/cgi-bin/singlerecord.cgi?id=hsa_circ_0005564) (5' ... 3')  [hsa-miR-644](http://www.mirbase.org/cgi-bin/mirna_entry.pl?acc=hsa-miR-644) (3' ... 5') | GUACGGCAGCAUCAACCACACAU                \|\|\|\|\|\|      CGAGAUUCUUUCGGUGUGA | 7mer-1a | 99 | 105 | 0.004 | 0.017 | -0.039 | 0.002 | -0.050 | -0.14 | 83 |
| [hsa_circ_0005564](http://www.circbase.org/cgi-bin/singlerecord.cgi?id=hsa_circ_0005564) (5' ... 3')  [hsa-miR-657](http://www.mirbase.org/cgi-bin/mirna_entry.pl?acc=hsa-miR-657) (3' ... 5') | AAGAUUGGCCCAGACAACCUGCC                  \|\|\|\|\|\|\|    GGAUCUCUCCCACUCUUGGACGG | 7mer-m8 | 289 | 295 | 0.003 | 0.032 | -0.059 | -0.004 | -0.032 | -0.18 | **90** |
| [hsa_circ_0005564](http://www.circbase.org/cgi-bin/singlerecord.cgi?id=hsa_circ_0005564) (5' ... 3')  [hsa-miR-657](http://www.mirbase.org/cgi-bin/mirna_entry.pl?acc=hsa-miR-657) (3' ... 5') | GACAAGGGCAACUACACCUGCAU        \|\|\|\|       \|\|\|\|\|\|   GGAUCUCUCCCACUCU--UGGACGG | 7mer-1a | 64 | 70 | -0.013 | 0.004 | -0.043 | -0.004 | -0.066 | -0.196 | **92** |
| [hsa_circ_0005564](http://www.circbase.org/cgi-bin/singlerecord.cgi?id=hsa_circ_0005564) (5' ... 3')  [hsa-miR-874](http://www.mirbase.org/cgi-bin/mirna_entry.pl?acc=hsa-miR-874) (3' ... 5') | UGGUGCCCUCUGACAAGGGCAAC      \|\|\|\|\|     \|\|\|\|\|\|   AGCCAGGGAGCCCGGUCCCGUC | 7mer-1a | 53 | 59 | -0.016 | 0.033 | -0.044 | 0.008 | -0.079 | -0.172 | 88 |
| [hsa_circ_0005564](http://www.circbase.org/cgi-bin/singlerecord.cgi?id=hsa_circ_0005564) (5' ... 3')  [hsa-miR-885-5p](http://www.mirbase.org/cgi-bin/mirna_entry.pl?acc=hsa-miR-885-5p) (3' ... 5') | CCACCUGGAGCAUCAUAAUGGAC                 \|\|\|\|\|\|    UCUCCGUCCCAUCACAUUACCU | 7mer-1a | 26 | 32 | 0.001 | 0.032 | -0.047 | -0.011 | 0.034 | -0.065 | 81 |
| [hsa_circ_0005564](http://www.circbase.org/cgi-bin/singlerecord.cgi?id=hsa_circ_0005564) (5' ... 3')  [hsa-miR-938](http://www.mirbase.org/cgi-bin/mirna_entry.pl?acc=hsa-miR-938) (3' ... 5') | GUGGUGCCCUCUGACAAGGGCAA                  \|\|\|\|\|\|     UGACCCAAGUGGAAAUUCCCGU | 7mer-1a | 52 | 58 | 0.001 | 0.013 | -0.044 | -0.012 | -0.063 | -0.179 | **92** |
| [hsa_circ_0005564](http://www.circbase.org/cgi-bin/singlerecord.cgi?id=hsa_circ_0005564) (5' ... 3')  [hsa-miR-940](http://www.mirbase.org/cgi-bin/mirna_entry.pl?acc=hsa-miR-940) (3' ... 5') | GAUUGGCCCAGACAACCUGCCUU                \|\|\|\|\|\|\|    CCCCUCGCCCCCGGGACGGAA | 7mer-m8 | 291 | 297 | 0.003 | -0.044 | -0.059 | 0.037 | -0.079 | -0.262 | **99** |
