# Supplementary material for: Circular RNA Circ_0005564 promotes osteogenic differentiation of bone marrow mesenchymal cells in osteoporosis
Source: Bioengineered. 2021 Aug 10;12(1):4911–23. doi: 10.1080/21655979.2021.1959865 (PMC8806437; doi:10.1080/21655979.2021.1959865)
Supplement: Supplemental Material [file KBIE_A_1959865_SM0129.zip › suppl/Table S3 new.docx]

Table S3. Circ_0005564-miRNA-mRNA interaction.

| circBase_ID | context+score percentile>=90 | Cumulative weighted context++ score<=-0.8 |
| --- | --- | --- |
| hsa_circ_0005564 | hsa-miR-940 | RPL37A |
| hsa_circ_0005564 | hsa-miR-940 | C15orf37 |
| hsa_circ_0005564 | hsa-miR-940 | MORN4 |
| hsa_circ_0005564 | hsa-miR-940 | SIRPG |
| hsa_circ_0005564 | hsa-miR-940 | COA3 |
| hsa_circ_0005564 | hsa-miR-940 | PRSS33 |
| hsa_circ_0005564 | hsa-miR-940 | APOBEC3F |
| hsa_circ_0005564 | hsa-miR-940 | C15ORF37 |
| hsa_circ_0005564 | hsa-miR-940 | SIRPB1 |
| hsa_circ_0005564 | hsa-miR-940 | SNRPD3 |
| hsa_circ_0005564 | hsa-miR-938 | TMEM151A |
| hsa_circ_0005564 | hsa-miR-938 | CTC-236F12.4 |
| hsa_circ_0005564 | hsa-miR-6893-5p | THEM5 |
| hsa_circ_0005564 | hsa-miR-6893-5p | DDA1 |
| hsa_circ_0005564 | hsa-miR-6893-5p | RBM23 |
| hsa_circ_0005564 | hsa-miR-6893-5p | IKBKG |
| hsa_circ_0005564 | hsa-miR-6893-5p | TNFRSF13C |
| hsa_circ_0005564 | hsa-miR-6893-5p | STMN2 |
| hsa_circ_0005564 | hsa-miR-6893-5p | KNCN |
| hsa_circ_0005564 | hsa-miR-6893-5p | AGO2 |
| hsa_circ_0005564 | hsa-miR-6893-5p | TFDP2 |
| hsa_circ_0005564 | hsa-miR-6893-5p | SUPT4H1 |
| hsa_circ_0005564 | hsa-miR-6893-5p | HHLA3 |
| hsa_circ_0005564 | hsa-miR-6893-5p | DYNLL2 |
| hsa_circ_0005564 | hsa-miR-6893-5p | NR6A1 |
| hsa_circ_0005564 | hsa-miR-6893-5p | RP11-664D7.4 |
| hsa_circ_0005564 | hsa-miR-6893-5p | C19orf54 |
| hsa_circ_0005564 | hsa-miR-6893-5p | CYP4A11 |
| hsa_circ_0005564 | hsa-miR-6893-5p | AC135178.1 |
| hsa_circ_0005564 | hsa-miR-6893-5p | MRPL12 |
| hsa_circ_0005564 | hsa-miR-6893-5p | C18orf21 |
| hsa_circ_0005564 | hsa-miR-6893-5p | S100A16 |
| hsa_circ_0005564 | hsa-miR-6893-5p | PHLDA3 |
| hsa_circ_0005564 | hsa-miR-6808-5p | FAM83F |
| hsa_circ_0005564 | hsa-miR-6808-5p | P2RY2 |
| hsa_circ_0005564 | hsa-miR-6808-5p | LHPP |
| hsa_circ_0005564 | hsa-miR-6808-5p | AL627309.1 |
| hsa_circ_0005564 | hsa-miR-6808-5p | GRINA |
| hsa_circ_0005564 | hsa-miR-6808-5p | FLJ45079 |
| hsa_circ_0005564 | hsa-miR-6808-5p | CYP4A22 |
| hsa_circ_0005564 | hsa-miR-6808-5p | UQCR11 |
| hsa_circ_0005564 | hsa-miR-6808-5p | APOC3 |
| hsa_circ_0005564 | hsa-miR-6808-5p | ABHD1 |
| hsa_circ_0005564 | hsa-miR-6808-5p | NDUFB4 |
| hsa_circ_0005564 | hsa-miR-6808-5p | PLAC8 |
| hsa_circ_0005564 | hsa-miR-6808-5p | ST8SIA2 |
| hsa_circ_0005564 | hsa-miR-6808-5p | TSPAN9 |
| hsa_circ_0005564 | hsa-miR-6808-5p | C14orf2 |
| hsa_circ_0005564 | hsa-miR-6808-5p | CCL22 |
| hsa_circ_0005564 | hsa-miR-6808-5p | THY1 |
| hsa_circ_0005564 | hsa-miR-6808-5p | ZNF581 |
| hsa_circ_0005564 | hsa-miR-6808-5p | FXYD2 |
| hsa_circ_0005564 | hsa-miR-6808-5p | LYRM4 |
| hsa_circ_0005564 | hsa-miR-6808-5p | CBX5 |
| hsa_circ_0005564 | hsa-miR-6808-5p | PACS2 |
| hsa_circ_0005564 | hsa-miR-6808-5p | TRIM44 |
| hsa_circ_0005564 | hsa-miR-6808-5p | C11orf48 |
| hsa_circ_0005564 | hsa-miR-6808-5p | CDK16 |
| hsa_circ_0005564 | hsa-miR-6808-5p | GATSL2 |
| hsa_circ_0005564 | hsa-miR-6808-5p | ZDHHC18 |
| hsa_circ_0005564 | hsa-miR-6808-5p | SELM |
| hsa_circ_0005564 | hsa-miR-657 | RPL30 |
| hsa_circ_0005564 | hsa-miR-657 | AGO2 |
| hsa_circ_0005564 | hsa-miR-657 | COPS2 |
| hsa_circ_0005564 | hsa-miR-657 | LSM3 |
| hsa_circ_0005564 | hsa-miR-657 | RBM23 |
| hsa_circ_0005564 | hsa-miR-657 | ZCCHC16 |
| hsa_circ_0005564 | hsa-miR-657 | MTRNR2L9 |
| hsa_circ_0005564 | hsa-miR-604 | TP73 |
| hsa_circ_0005564 | hsa-miR-604 | FAM109B |
| hsa_circ_0005564 | hsa-miR-604 | ABHD6 |
| hsa_circ_0005564 | hsa-miR-604 | HCFC1 |
| hsa_circ_0005564 | hsa-miR-604 | ST3GAL3 |
| hsa_circ_0005564 | hsa-miR-1322 | MTO1 |
| hsa_circ_0005564 | hsa-miR-1231 | CDR1as |
| hsa_circ_0005564 | hsa-miR-1231 | CREB3L3 |
| hsa_circ_0005564 | hsa-miR-1231 | NBPF1 |
| hsa_circ_0005564 | hsa-miR-1231 | SCGB2A2 |
| hsa_circ_0005564 | hsa-miR-1231 | C12orf57 |
| hsa_circ_0005564 | hsa-miR-1208 | DR1 |
| hsa_circ_0005564 | hsa-miR-1208 | CYBRD1 |
| hsa_circ_0005564 | hsa-miR-1208 | OSBPL2 |
