# Supplementary figures and images for: Circular RNA Circ_0005564 promotes osteogenic differentiation of bone marrow mesenchymal cells in osteoporosis
Source: Bioengineered. 2021 Aug 10;12(1):4911–23. doi: 10.1080/21655979.2021.1959865 (PMC8806437; doi:10.1080/21655979.2021.1959865)

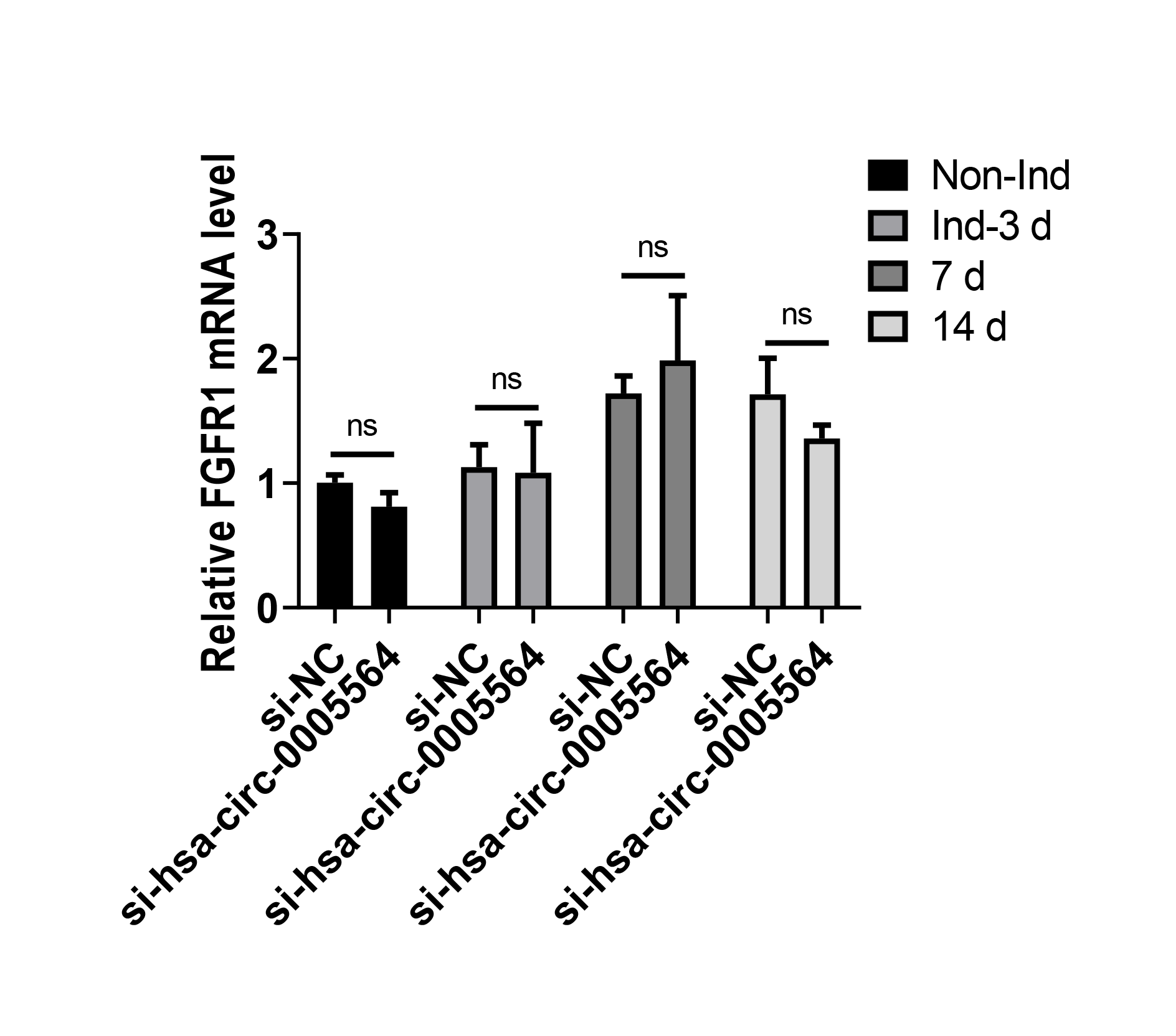

Supplement: Supplemental Material [file KBIE_A_1959865_SM0129.zip › suppl/Figure S1 new.tif]
